# The use of the induced membrane technique in an open fracture of the first phalangeal column of the thumb

**DOI:** 10.1016/j.tcr.2025.101203

**Published:** 2025-05-26

**Authors:** Sohayb Darraz, Amine El Farhaoui, Mohammed Lamziraa, Llyesse Haichour, Omar Mokhtari, Adnane Lachkar, Najib Abdeljaouad, Hicham Yacoubi

**Affiliations:** Faculty of Medicine and Pharmacy, Mohammed Ist University, Oujda, Morocco; Department of Traumatology, orthopedic Mohammed VI University Hospital Mohammed I University Oujda, Morocco; Mohammed First University, Faculty of Medecine and Pharmacy, LAMCESM, Oujda, Morocco

**Keywords:** Open fracture, Induced membrane, Tendon lesions, Bone loss

## Abstract

The case involves a 53-year-old patient admitted for trauma to the right hand following an accident with a grinder. The 4 cm wound on the first ray caused a tendon deficit without vascular or nerve damage. An open fracture of the first metacarpal with a 2 cm bone loss was diagnosed. Initial treatment included wound irrigation, debridement, suturing, and osteosynthesis using biological cement and a Bone fixation using Iselin pinning with two K-wires. A second procedure, six weeks later, involved a bone graft using the Masquelet technique, showing bone consolidation after five months. This technique is proving increasingly effective for complex open fractures, promoting vascularization and cortication through the release of growth factors like BMP-2 and VEGF. Studies confirm the presence of osteoclasts and osteoblasts in the induced membrane, aiding osteointegration. Initially developed for the lower limb, the technique has successfully been extended to the hand, providing a viable option for bone substance loss.

## Introduction

The limitations of bone grafts have been overcome thanks to the mechanical and biological advantages of pseudo-synovial membranes that the body forms around foreign objects. The induced membrane technique for reconstructing long bone defects was first described in 2000 in a series of 35 cases [1].

Managing open fractures with significant bone loss remains a challenge for surgeons. A method developed by Masquelet and colleagues [[2], [3], [4], [5]] involves two distinct surgical steps. The concept is to fill the bone defect with an inert material, such as cement, to create a membrane around it, similar to one formed around a foreign object. This membrane forms a chamber that, once the cement is removed, can host a cancellous bone graft. Initially thought to act as a protective barrier against bone resorption, this technique is increasingly used for open fractures with bone substance loss.

## Case presentation

Our patient, a 53-year-old male with type 2 diabetes controlled by oral antidiabetic drugs, was admitted following a trauma to the right hand's first ray caused by a grinder accident.

**Fig. 1:**
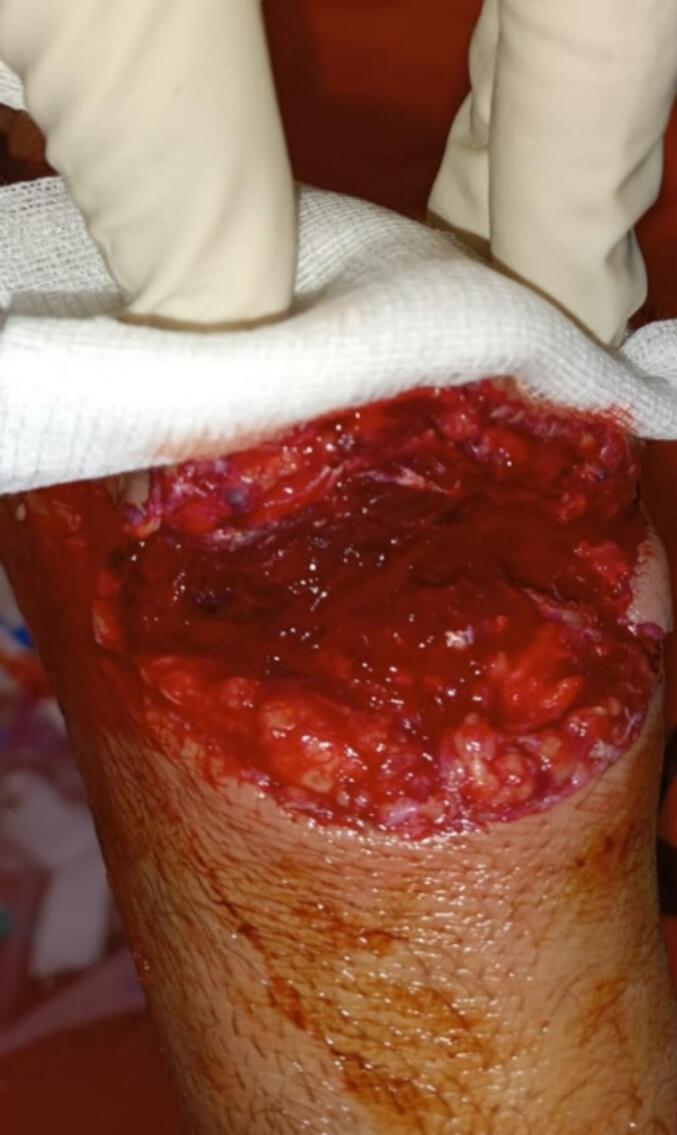
Clinical image of the wound upon admission.

On clinical examination, the patient was conscious, hemodynamically and respiratory stable, with a 4 cm circumferential wound on the dorsal side of the first ray of the right hand (Fig. 1). Tendon examination revealed a deficit in thumb extension and abduction. The vascular and nerve examination showed no abnormalities.

**Fig. 2:**
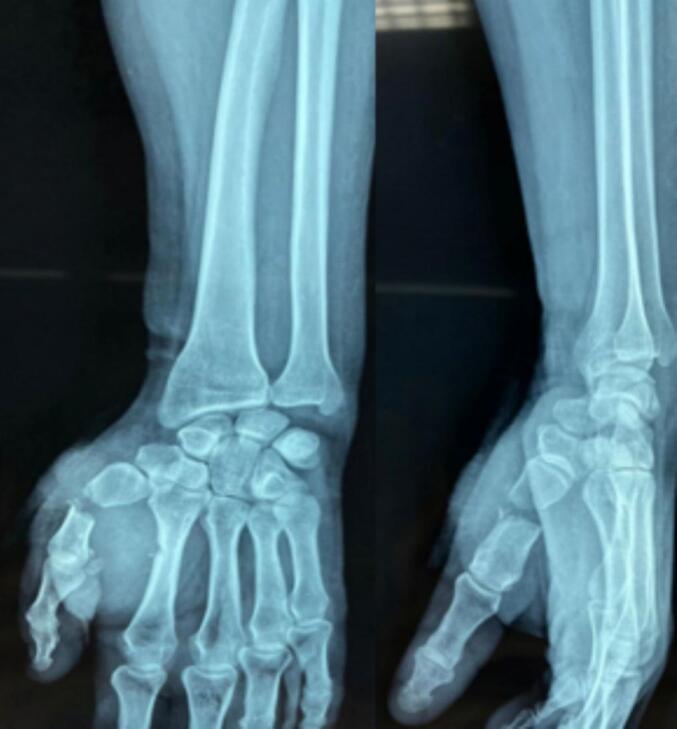
Radiographic image showing a diaphyseal fracture of the first metacarpal with bone substance loss.

The patient underwent wound irrigation and debridement 5 h after the trauma, followed by wound suturing. Standard radiography revealed an open fracture of the first metacarpal, classified as stage 2 according to both the Cauchoix-Duparc and Gustilo classifications, with an estimated 2 cm bone loss (Fig. 2). The patient was taken to the operating room 12 h after admission.

In the operating room, the first step involved osteosynthesis of the first metacarpal using the Islen technique, along with the application of biological cement to fill the bone defect.

**Fig. 3:**
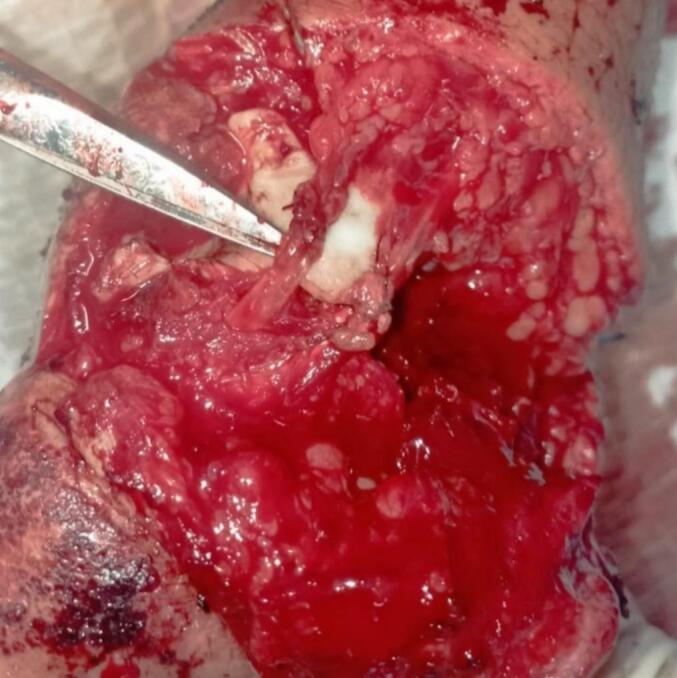
Intraoperative image of the tendon suturing of the extensor pollicis brevis and abductor pollicis longus.

**Fig. 4:**
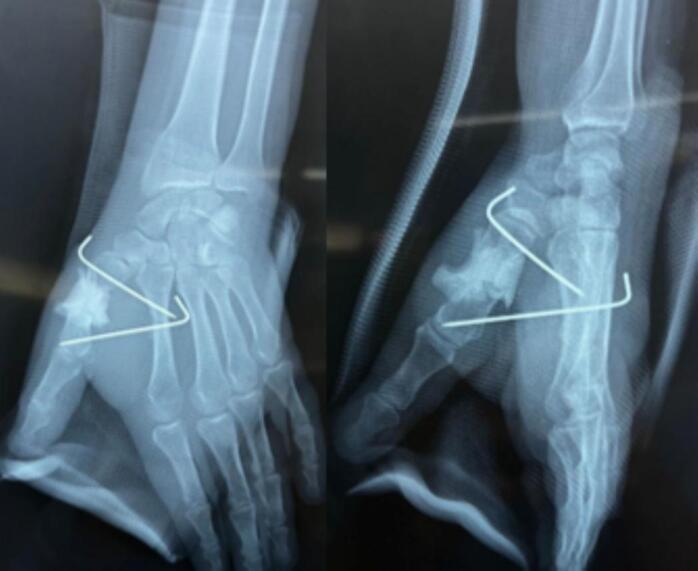
Postoperative radiograph of the first stage of the Masquelet procedure.

The second step consisted of exploring the wound, revealing a complete rupture of the extensor pollicis brevis and the abductor pollicis longus. Tendon repair was performed using the Kessler technique (Fig. 3). A splint was applied, and the patient received antibiotic therapy for 48 h with a combination of 2 g amoxicillin/clavulanic acid and gentamicin, along with a control X-ray (Fig. 4).

**Fig. 5:**
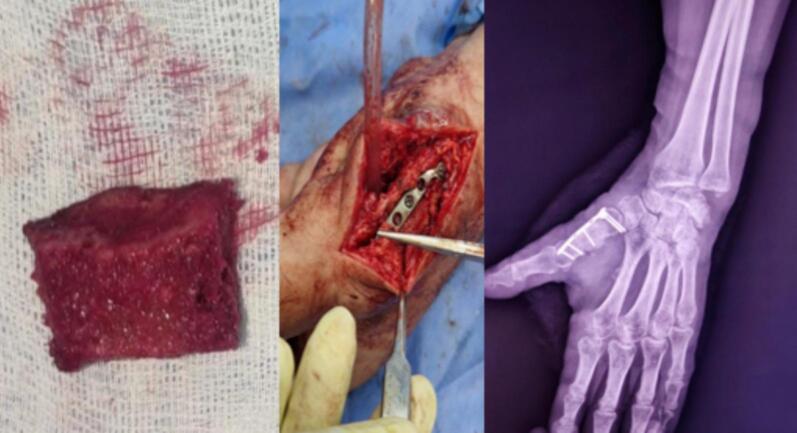
Intraoperative images and postoperative radiograph of the second stage of the Masquelet procedure.

Six weeks later, the patient was readmitted for the second stage of the Masquelet technique. In the operating room, a bone graft was harvested from the anterior iliac crest, followed by a dorsal thumb incision and opening of the induced membrane. The cement was removed in one piece, and the bone graft was placed, secured with a mini-plate for osteosynthesis (Fig. 5).

**Fig. 6:**
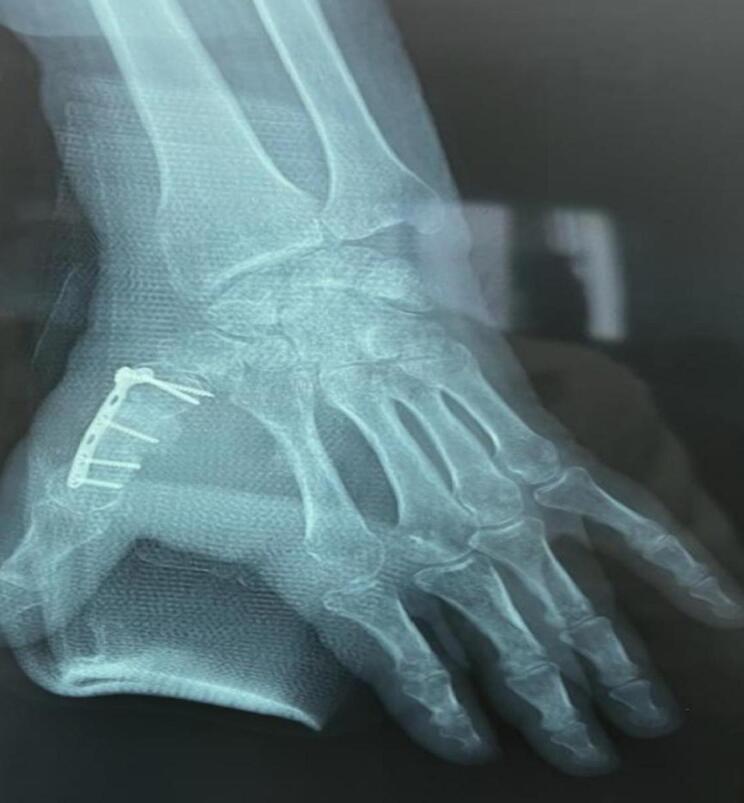
X-ray at the 6-week follow-up of the second stage of the Masquelet procedure, showing good bone consolidation.

Follow-up visits every three weeks showed proper wound healing and good progress in thumb extension and adduction (Fig. 6). At the 5-month check-up, the bone had consolidated, and the hand was fully functional.

The effectiveness of the induced membrane technique in managing open fractures, even those exceeding the 6-h window, is becoming increasingly recognized. In our patient's case, the open fracture was successfully managed without complications, thanks to this technique.

## Discussion

Our case reports a bone defect in the first column of the thumb following an open fracture, successfully managed using the induced membrane technique. This result highlights the effectiveness of the Masquelet technique for reconstructing bone loss in the hand, including infected cases and without limitations on the defect size.

Various studies have explored the physiology of the induced membrane generated through the Masquelet method. This membrane plays a crucial role in preventing bone graft resorption while promoting vascularization and bone cortication through the release of growth factors such as vascular endothelial growth factor (VEGF), which directly impacts osteoinductive elements like bone morphogenetic protein (BMP-2) [6]. A recent study by Gouron and colleagues [7] revealed the simultaneous presence of calcitonin receptors and mononuclear cell activators in this induced membrane, confirming the presence of osteoclasts and osteoblasts, suggesting its potential to enhance the osteointegration of bone grafts. While the optimal time interval between the first and second stages of the procedure has not been clearly defined, histological analyses have shown that the induced membrane reaches its peak BMP-2 production around the fourth week after cement placement [6].

The induced membrane technique, conceived and developed by Masquelet, offers new possibilities for addressing bone defects. Pelissier et al. were the first to experimentally demonstrate the biological significance of this membrane [6]. Pelissier showed that, in the presence of cellular debris, there is cellular proliferation and differentiation into the bone lineage, which he attributed to the presence of VEGF, TGF-beta 1, and BMP-2 in the membrane.

The induced membrane technique has been an additional option we have used for several years to treat bone defects, not only in the lower limb but also in the hand and wrist [[8], [9], [10]].

The protective role of the membrane over the cancellous bone graft and its ability to produce growth factors beneficial for bone consolidation were revealed through laboratory research by Pelissier et al. [11,12]. Only more recently have its indications been extended to hand trauma surgery, with results that have impressed practitioners. In a recent series, Flamans et al. [13]. reported 11 bone grafts using the induced membrane technique in the hand. Of these cases, eight were due to trauma, and three resulted from nonunion. Nine consolidations were achieved on average by the fourth month (ranging from 3 to 12 months).

The discovery of stem cells within the membrane [[14], [15], [16]] has made the previously recommended 4 to 6-week interval less critical [17].

Recent research [18] has indicated that antibiotics present in the cement alter the physical characteristics of the membrane, although the impact on bone formation has not been clearly defined. It is also likely that the properties of the spacer components influence the characteristics of the membrane [19].

## Conclusion

This case demonstrates the successful use of the Masquelet technique in managing a complex open fracture with significant bone loss in the first ray of the hand. The technique, initially developed for long bones in the lower limb, has proven effective for hand injuries, promoting bone consolidation through the release of growth factors like BMP-2 and VEGF. The induced membrane plays a critical role in preventing graft resorption and supporting osteointegration by fostering the presence of osteoclasts and osteoblasts. This approach offers a reliable and viable solution for addressing bone substance loss in complex fractures, even in the hand.

## CRediT authorship contribution statement

**Sohayb Darraz:** Writing – original draft, Methodology, Formal analysis, Data curation. **Amine El Farhaoui:** Data curation. **Mohammed Lamziraa:** Data curation. **Llyesse Haichour:** Data curation. **Omar Mokhtari:** Data curation. **Adnane Lachkar:** Supervision. **Najib Abdeljaouad:** Supervision. **Hicham Yacoubi:** Supervision.

## Declaration of competing interest

The authors declare that they have no known competing financial interests or personal relationships that could have appeared to influence the work reported in this paper.
